# Supplementary material for: Enhancement of hMSC In Vitro Proliferation by Surface Immobilization of a Heparin-Binding Peptide
Source: Molecules. 2023 Apr 13;28(8):3422. doi: 10.3390/molecules28083422 (PMC10146743; doi:10.3390/molecules28083422)
Supplement: Supplementary file 1 [file molecules-28-03422-s001.zip › molecules-2282003-supplementary.pdf]

Supporting information

Enhancement of hMSC In Vitro Proliferation by Surface Immobilization of a Heparin-Binding Peptide

Maura Cimino [1,2,3], Paula Parreira [1,2], Victoria Leiro [1,2], Aureliana Sousa [1,2], Raquel M. Gonçalves [1,2,4], Cristina C. Barrias [1,2,4] and M. Cristina L. Martins [1,2,4,*]

1 i3S—Instituto de Investigação e Inovação em Saúde, Rua Alfredo Allen, 208, 4200-135 Porto, Portugal
2 INEB—Instituto de Engenharia Biomédica, Rua Alfredo Allen, 208, 4200-135 Porto, Portugal
3 Faculdade de Engenharia, Departamento de Engenharia Metalúrgica e de Materiais, Universidade do Porto, Rua Dr. Roberto Frias, 4200-465 Porto, Portugal
4 ICBAS—Instituto de Ciências Biomédicas Abel Salazar, Universidade do Porto, Rua de Jorge Viterbo Ferreira, 228, 4050-313 Porto, Portugal
* Correspondence: cmartins@ineb.up.pt

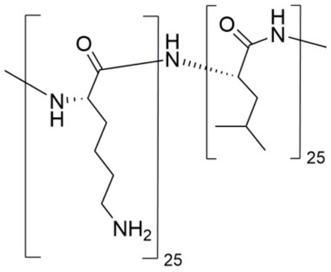

Figure S1. Schematic representation of the block co-polypeptide poly(L-lysine, L-leucine) (pKL) synthesized via a ring-opening polymerization of the corresponding amino acid N-carboxyanydrides (NCAs):

i) **Synthesis of the copolypeptide poly(N-ε-benzyloxycarbonyl-L-lysine)25-ran-poly(L-leucine)25 (p(Z)KL).** N-ε-benzyloxycarbonyl-L-lysine-N-carboxyanhydride (Z-Lys NCA) (3.5 g, 11.0 mmol) and L-leucine-N-carboxyanhydride (Leu NCA) (1.8 g, 11.0 mmol) were independently dissolved in dry THF (100 mL and 50 mL, respectively) and introduced in a 250 mL round bottom flask with stir bar. A tert-butyl amine initiator solution (33 mg in 5 mL of THF) was then added. The reaction mixture was stirred under inert atmosphere (Argon) for 24 h at 22ºC. After this time, an aliquot (100 µL) was removed and analyzed by FTIR to confirm that all the Z-Lys NCA and Leu NCA were consumed. Once confirmed, the copolypeptide was precipitated by adding methanol (500 mL), and then isolated by centrifugation. The copolymer pellet was then soaked in methanol (50 mL) for 2 h, and then centrifugated again to render the protected copolymer. After drying under vacuum overnight, a white powder was obtained (3.68 g, 79% yield).

ii) **Deprotection of Cbz-Lysine: Poly(L-lysine-HBr)25-ran-poly(L-leucine)25 (pKL).** The protected copolypeptide p(Z)KL (3.6 mg) was introduced in a 100 mL round-bottom flask and TFA was added (5 mL). The flask was placed in an ice bath and allowed to stir for 15 min, to allow the copolymer to dissolve. After that, with the mixture at 0ºC, HBr (8 mL of a 33% solution in HOAc) was added dropwise and the solution was then allowed to stir in the ice bath for 3 h. After this time, diethyl ether (200 mL) was added to precipitate the product. The mixture was centrifuged to isolate the solid precipitate, and the product was subsequently washed with diethyl ether (100 mL), several times until yield a white solid. After drying the sample under vacuum, the precipitated was resuspended in water and the solution was placed in a dialysis bag (2000 Da MWCO). The sample was dialyzed against deionized water for 3 days (water changed every 8 h). After dialysis, the sample was lyophilized to give the product as a white fluffy powder (2.98 g, 77% yield).

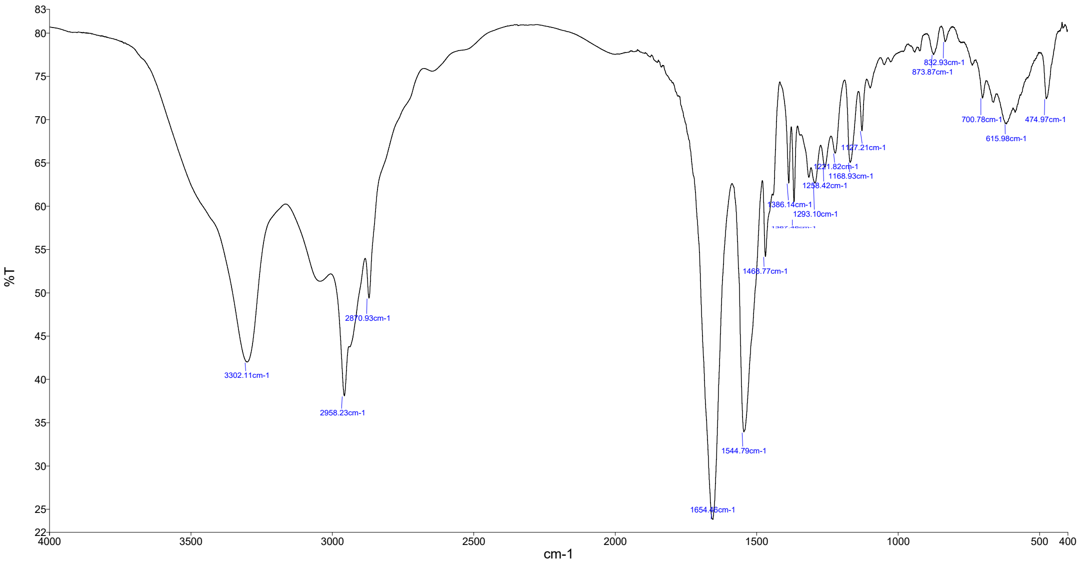

Figure S2. Fourier-transform infrared spectroscopy (FTIR) spectrum of pKL in the 2000 cm^{-1}- 400 cm^{-1} region.

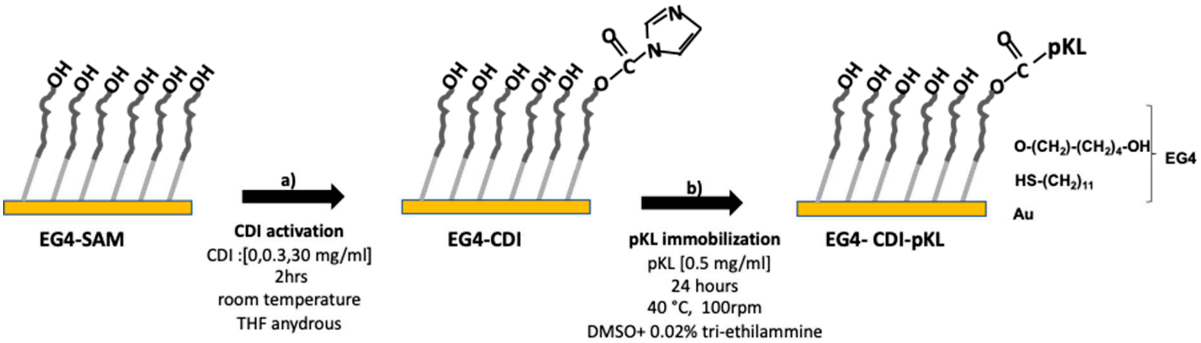

Figure S3. pKL covalent immobilization on EG4-SAMs. a) activation of -OH groups at EG4-SAMs via CDI chemistry; b) pKL conjugation on CDI-activated EG4-SAMs through its NH2.

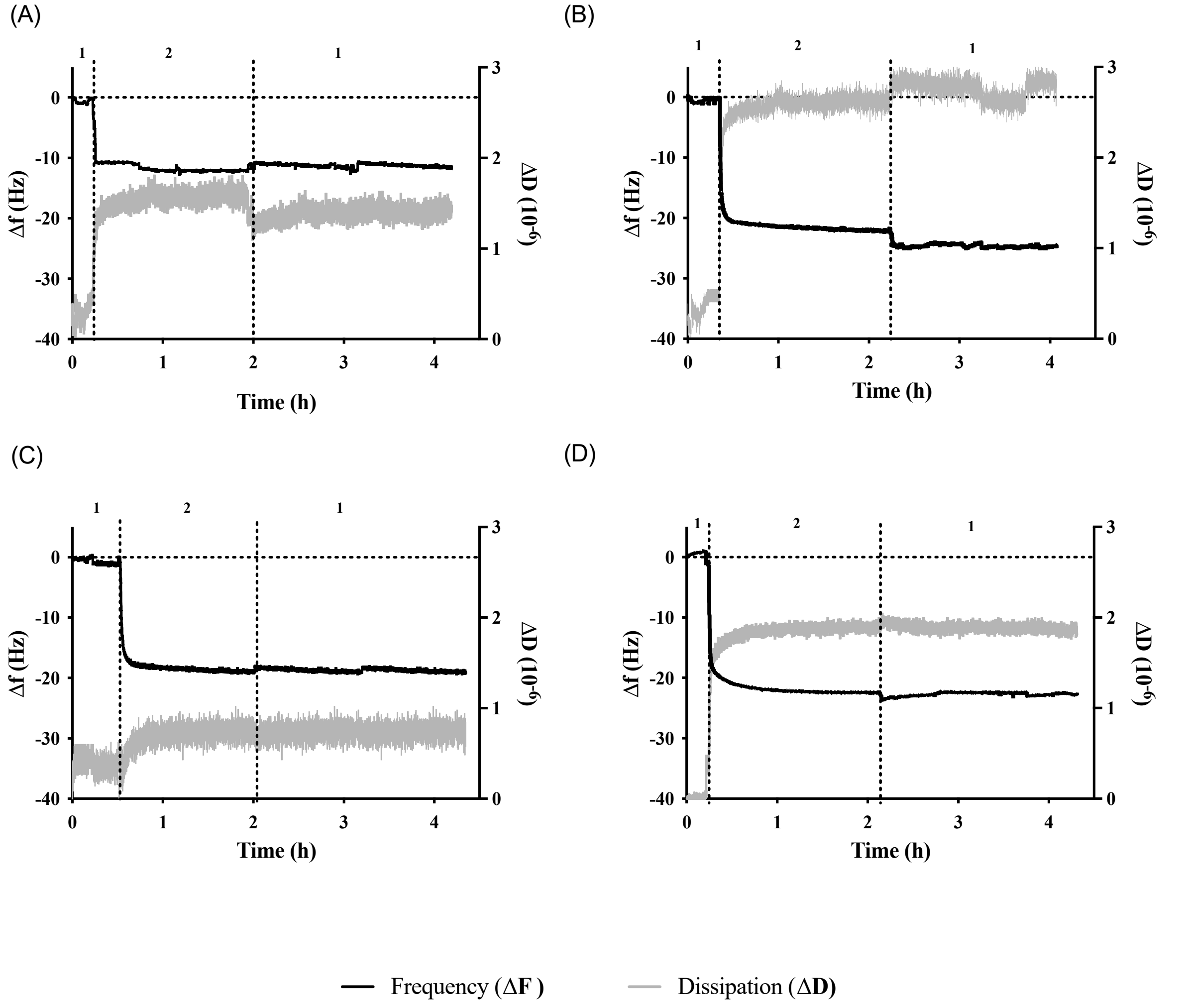

Figure S4. Representative time-resolved QCM-D measurement parameter shifts, revealing the characteristic Frequency and Dissipation profiles from the seventh overtone in response to interactions between heparin and: (A) 0.3 CDI-buffer, (B) 0.3 CDI-pKL, (C) 30 CDI-buffer, (D) 30 CDI-pKL. Dotted vertical lines represent the injection on the system of: 1- PBS (0.1M); 2- heparin [0.1 mg/mL].

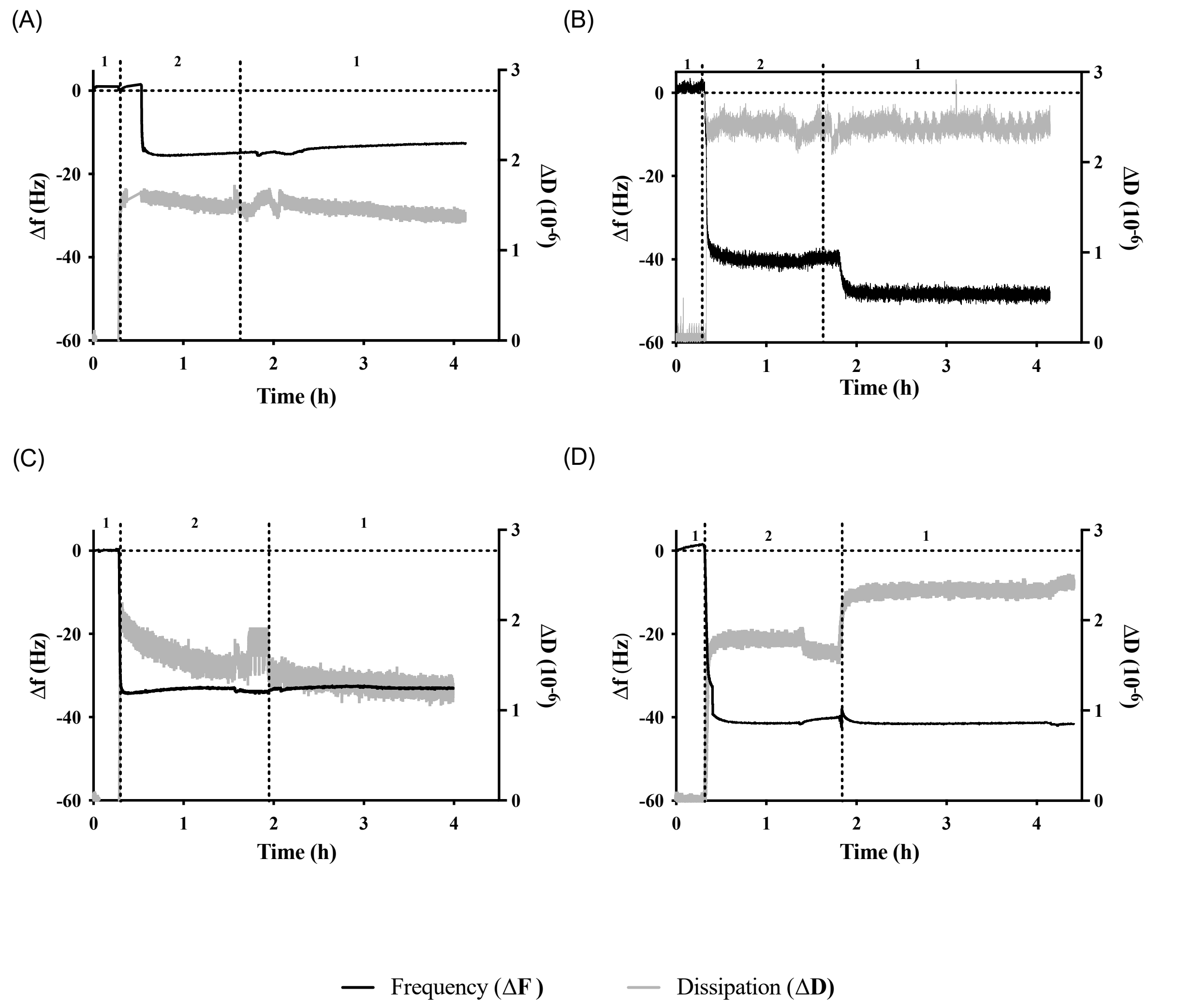

Figure S5. Representative time-resolved QCM-D measurement parameter shifts, revealing the characteristic Frequency and Dissipation profiles from the seventh overtone in response to interactions between fibronectin and: (A) 0.3 CDI-buffer, (B) 0.3 CDI-pKL, (C) 30 CDI-buffer, (D) 30 CDI-pKL. Dotted vertical lines represent the injection on the system of: 1- PBS (0.1M); 2- fibronectin [40 μg/mL].

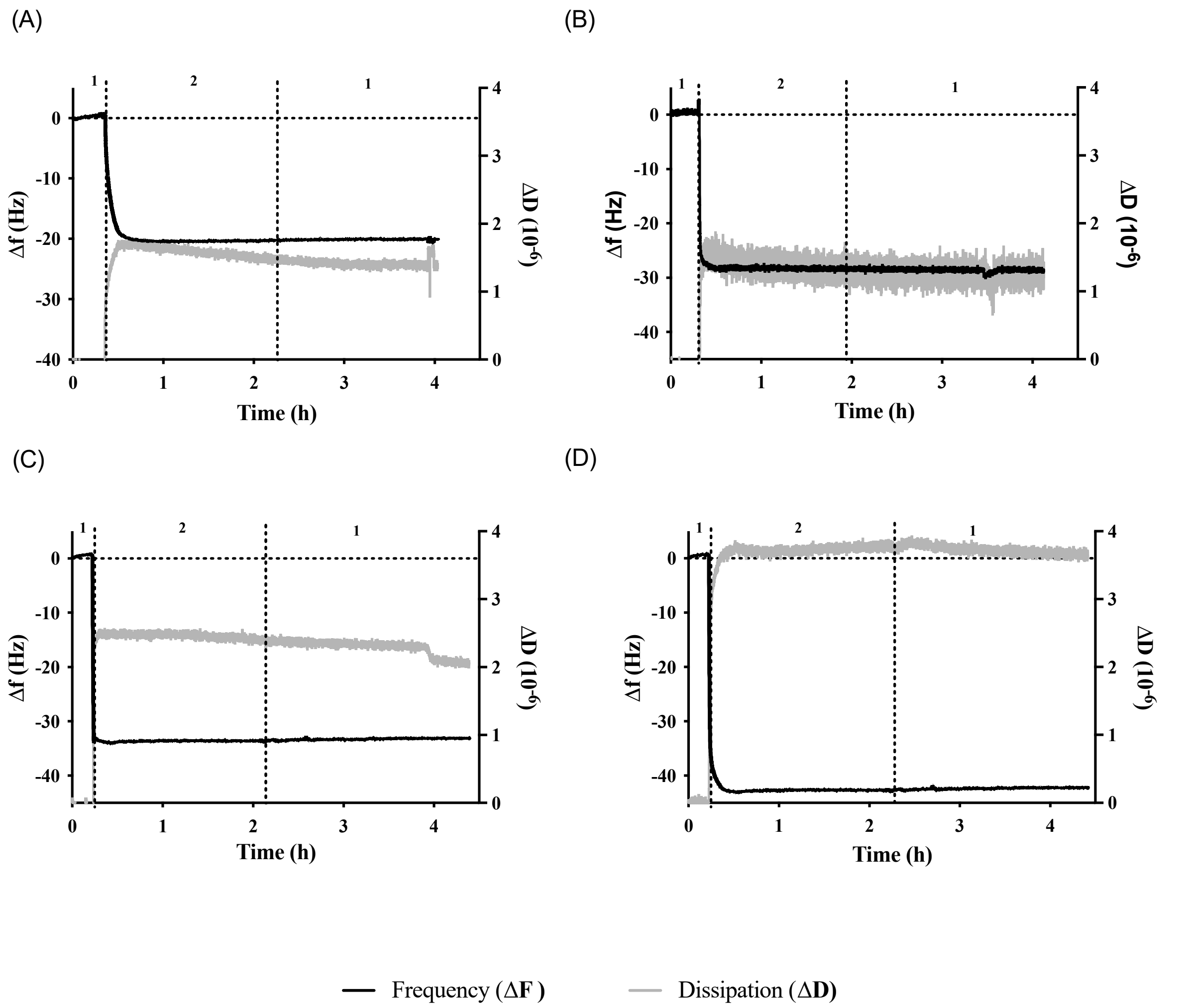

Figure S6. Representative time-resolved QCM-D measurement parameter shifts, revealing the characteristic Frequency and Dissipation profiles from the seventh overtone in response to interactions between FBS and: (A) 0.3 CDI-buffer, (B) 0.3 CDI-pKL, (C) 30 CDI-buffer, (D) 30 CDI-pKL. Dotted vertical lines represent the injection on the system of: 1- PBS (0.1M); 2- 1% FBS.
